# Pain catastrophizing in rheumatic diseases: prevalence, origin, and implications

**DOI:** 10.1007/s00296-024-05583-8

**Published:** 2024-04-12

**Authors:** Mateusz Wilk, Olena Zimba, Glenn Haugeberg, Mariusz Korkosz

**Affiliations:** 1 Division of Rheumatology, Immunology and Internal Medicine, University Hospital in Krakow, Krakow, Poland; 2 National Institute of Geriatrics, Rheumatology and Rehabilitation, Warsaw, Poland; 3 Department of Internal Medicine N2, Danylo Halytsky Lviv National Medical University, Lviv, Ukraine; 4 Division of Rheumatology, Department of Internal Medicine, Sørlandet Hospital, Kristiansand, Norway; 5 Department of Neuromedicine and Movement Science, Faculty of Medicine and Health Sciences, NTNU, Norwegian University of Science and Technology, Trondheim, Norway; 6 Department of Rheumatology and Immunology, Jagiellonian University Medical College, Świętej Anny 12 St., 31-008 Kraków, Poland

**Keywords:** Pain, Catastrophization, Depression, Review, Musculoskeletal diseases

## Abstract

Pain is a crucial factor in rheumatic disorders, and reducing it is a primary goal of successful treatment. Adaptive pain-coping strategies can enhance this improvement, but maladaptive approaches such as pain catastrophizing may worsen overall patient well-being. This narrative review aims to provide a concise overview of the existing knowledge on pain catastrophizing in the most prevalent specific rheumatic disorders. The objective of this study was to improve understanding of this phenomenon and its implications, as well as to pinpoint potential directions for future research. We conducted searches in the MEDLINE/PubMed, SCOPUS, and DOAJ bibliography databases to identify articles related to pain catastrophizing in rheumatoid arthritis, psoriatic arthritis, axial spondylarthritis, systemic sclerosis, systemic lupus erythematosus, Sjögren’s syndrome, juvenile idiopathic arthritis, and osteoarthritis (non-surgical treatment). Data extraction was performed on November 1, 2023. The investigators screened the identified articles to determine their relevance and whether they met the inclusion criteria. Following a bibliography search, which was further expanded by screening of citations and references, we included 156 records in the current review. The full-text analysis centred on pain catastrophizing, encompassing its prevalence, pathogenesis, and impact. The review established the role of catastrophizing in amplifying pain and diminishing various aspects of general well-being. Also, potential treatment approaches were discussed and summarised across the examined disorders. Pain catastrophizing is as a significant factor in rheumatic disorders. Its impact warrants further exploration through prospective controlled trials to enhance global patient outcomes.

**Supplementary Information:**

The online version contains supplementary material available at 10.1007/s00296-024-05583-8.

## Introduction

Rheumatic disorders contribute substantially to morbidity and healthcare expenses on a global scale [1–3]. Their distinguishing feature is variability in pain levels. Treatment strategies primarily target the underlying causes of pain, which often stems from inflammation or degenerative processes. Pain can be debilitating by diminishing functionality and compromising the quality of life across different aspects [4]. Consequently, it is imperative to recognise pain as a fundamental aspect and a critical determinant of treatment outcomes, particularly from the patient's viewpoint [5].

Over the past few decades, a range of theories on the origin of pain have surfaced in the scientific discourse. The landscape of understanding continues to expand as new pathways are uncovered through scientific inquiry. Notably, a biopsychosocial model of pain is well recognised as a possible hypothesis [6]. Consequently, these dimensions are being investigated to identify additional factors related to pain; to refine treatment approaches; and to enhance outcomes associated with pain.

Given the well-established recognition of mood and depression disorders as potential contributors to altered pain perception, ongoing investigation seeks to pinpoint the specific causes of intensified pain. One particularly intriguing factor is pain catastrophizing, a psychological maladaptive coping mechanism linked to an exaggerated experience of pain. This phenomenon is characterised by three primary domains: helplessness, rumination, and magnification.

In recent years, there have been concerted efforts to thoroughly investigate catastrophizing across various entities [7, 8]. While some authors have delved into the general phenomenon of pain catastrophizing, a detailed discussion of it is omitted here to maintain the focus of the current narrative review article [9, 10]. Additionally, prior review articles on pain catastrophizing in rheumatic disorders may not encompass recent discoveries from the last few years. These reviews may also not specifically address the most crucial rheumatic musculoskeletal disorders separately and distinctly.

In addition to elucidating the current understanding of pain catastrophizing in distinct rheumatic disorders, this narrative review aims to delve into unexplored dimensions that could improve diagnostic and treatment approaches. Beyond describing existing knowledge, we seek to unearth subtle nuances and potential intersections with other factors that may modify the impact of pain catastrophizing on rheumatic disorders.

In the broader context, our aim is to catalyse a shift in the clinical mindset to foster increased awareness among healthcare professionals about the pivotal role of pain catastrophizing in rheumatic disorders. By emphasising this aspect, we hope to inspire clinicians to proactively incorporate assessments and interventions related to pain catastrophizing into their routine practice, thereby optimizing patient care and outcomes. Using this approach, we hope to contribute to the advancement of both theoretical frameworks and practical strategies that can ultimately enhance the quality of care provided to individuals grappling with these complex conditions.

## Pain catastrophizing

Pain catastrophizing is a psychological response to pain characterised by an exaggerated perception of the pain's threat and a sense of helplessness in managing it. Those who engage in pain catastrophizing often anticipate the worst outcomes, ruminate on pain-related thoughts, and expect their pain to persist or intensify. This cognitive and emotional reaction can contribute to increased distress, amplified pain sensitivity, and challenges in coping with and adapting to pain.

Albert Ellis and later Aaron Beck conceptualised catastrophizing as a maladaptive cognitive style utilised by individuals experiencing anxiety and depressive disorders [11, 12]. The early stages of study encountered challenges due to the absence of effective instruments for measuring and comparing catastrophizing. Addressing this limitation, Rosentiel et al. [13] introduced the Coping Strategies Questionnaire (CSQ), which focused on one dimension of catastrophizing—helplessness in the context of pain[13]. Following this, Sullivan et al. [14] formulated the Pain Catastrophizing Scale (PCS), a universal and multidimensional instrument designed to comprehensively measure and explore catastrophizing, encompassing its primary domains of magnification, rumination, and helplessness [14]. When comparing the CSQ and PCS, it became evident that using CSQ alone would not assess certain crucial domains of catastrophizing. These scales have proven to be valuable tools: they have been consistently applied and validated across diverse populations and diagnostic categories, including in rheumatic musculoskeletal disorders [15–17].

## Search methodology

We conducted a literature search in the MEDLINE/PubMed, Scopus, and Directory of Open Access Journals (DOAJ) databases using terms associated with pain catastrophizing and specific diagnoses. The literature search involved predefined word strings for each disease (see Appendix) in PubMed and Scopus. Furthermore, as DOAJ does not offer an advanced search engine, the search was carried out using the basic term [disease] + catastrophizing.

The search and extraction of the primary article pool took place on November 1, 2023. Subsequent processing of articles, including title, abstract, full-text screening, as well as citation and reference screening, was performed between November 1 and December 13, 2023. The reference list has been updated to reflect the most current information as of the submission date (March 5, 2024).

**Fig. 1:**
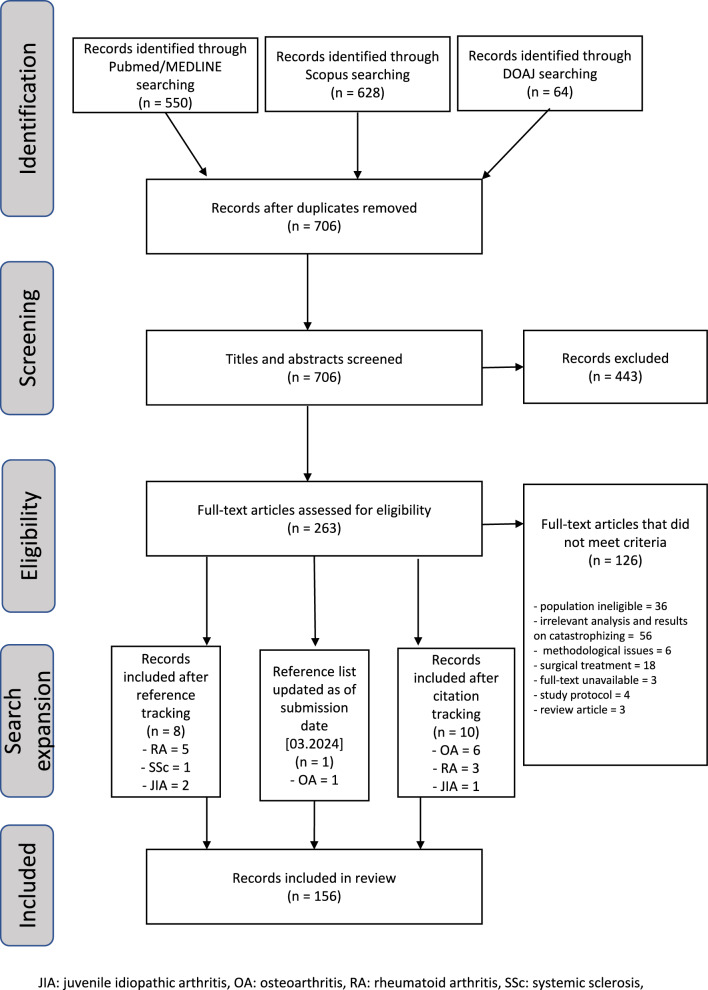
Literature search flowchart

Our search yielded 550 MEDLINE/PubMed-indexed articles, 628 Scopus-indexed articles, and 64 DOAJ-indexed articles. After eliminating duplicates, a total of 706 articles remained. One author (MW) conducted screening based on article abstracts and titles. Following the initial screening, 263 full-text articles were further evaluated for eligibility. The inclusion of additional articles from reference screening, citation screening, and from additional search as of the submission date led to the final inclusion of 156 articles in the review. The literature selection flowchart is depicted in Fig. 1.

The inclusion criteria were as follows:

1. Full-text availability.
2. English language articles.
3. Articles assessing catastrophizing, either as a primary or secondary focus, separately across the examined diagnoses. These diagnoses included rheumatoid arthritis (RA), psoriatic arthritis (PsA), axial spondylarthritis (AxSpA), systemic lupus erythematosus (SLE), systemic sclerosis (SSc), Sjögren’s syndrome (SS), juvenile idiopathic arthritis (JIA), and conservatively treated osteoarthritis (OA).
4. Use of validated and accepted instruments to measure catastrophizing, such as the CSQ and PCS, and derivates.

The exclusion criteria were as follows:

1. Ineligible population groups, such as combined cohorts that would not allow us to draw specific conclusions about distinct diagnostic groups. Vague cohorts where specific diagnosis information was uncertain, such as those labelled as "knee pain", were also excluded.
2. Studies with irrelevant analyses and results on catastrophizing. This criterion applied when a study did not yield any specific or valuable conclusions regarding pain catastrophizing in rheumatic disorders, primarily because the study did not focus on pain catastrophizing or employed alternative methods to assess catastrophizing.
3. Methodological issues leading to a perceived high risk of biased results which could compromise the reliability and quality of the review.
4. Studies involving surgical treatment, as the review focused on conservative treatment, aligning with ordinary rheumatology practices. Consequently, studies related to pain catastrophizing as a variable associated with surgical treatment (e.g., arthroplasty) were excluded. It is noteworthy that numerous meta-analyses and systematic reviews addressing surgical treatments in such cases have already been conducted [18–21].
5. Preprints, commentaries, case studies, errata, study protocols, and reviews or meta-analyses were not included in our study.

There were no restrictions on the publication date of included articles.

The concept of pain catastrophizing was examined individually within major rheumatic disorders. Initially, our focus was directed towards illustrating the potential prevalence and intensity of catastrophizing across these diseases. We highlighted demographic, psychosocial, and clinical factors from the current literature that could potentially be associated with catastrophizing. We also underscored the implications of pain catastrophizing and discussed potential treatment approaches.

Throughout the manuscript, the terms "pain catastrophizing" and "catastrophizing" are used interchangeably as synonyms to enhance the readability of this review article.

The methodology employed in our search process and the subsequent development of this narrative review was guided by previously published articles on the subject [22–25].

### Rheumatoid arthritis

The initial search identified 132 potentially eligible articles. After removing duplicates and screening abstracts and titles, 45 articles remained. Further examination during full-text screening resulted in 31 articles meeting the criteria for inclusion in the review. Additionally, five articles were found through reference tracking, and three more through citation tracking, bringing the total number of eligible articles in the current review to 39.

The prevalence of pain catastrophizing appears to be relatively high among RA patients, who were also were found to engage in catastrophizing behaviours significantly more often than healthy individuals [26]. A direct comparison between major rheumatic disorders has been conducted previously [27]. It utilised a simplified approach to assess pain catastrophizing with a scale consisting of 2 questions instead of the original 13 questions, resulting in a score of 0–6 points [28]. RA patients had a mean simplified PCS score of 1.88. While no significant differences between RA and PsA patients were noted, patients with axSpA exhibited significantly higher catastrophizing behaviours than RA counterparts. When comparing the proportion of individuals classified as high pain catastrophizers (the authors of that study used an arbitrary cut-off score of ≥ 4), there were no significant differences among RA (10.5%), PsA (12.7%), and axSpA (15.3%) patients. In a distinct analysis, despite a modest elevation in PCS scores within the axSpA group as compared to the RA group, this observed difference was not statistically significant (20.8 vs. 17.0, *p* = 0.08) [29]. An additional direct comparison of RA and SSc patients also showed no significant difference in mean PCS scores [30].

Multiple factors associated with higher levels of catastrophizing were identified across the explored analyses, including increased global pain, impaired physical function, and lower perceived general health status [27]. The evidence on the influence of age on catastrophizing appears to be conflicting. Some articles suggest that younger age may be linked to greater catastrophizing, with the hypothesis that coping mechanisms may develop and improve over time [27]. In contrast, another analysis found that catastrophizing is more prevalent among older adults experiencing mild RA pain [31]. However, as the pain reaches a severe level, age differences in coping strategies diminish. Authors suggest that older adults better comprehend the challenges of managing severe pain and employ more effective coping responses by drawing from their experiences. Other analyses have suggested that completing fewer years of education contributes to greater catastrophizing [27, 32, 33].

Several psychological factors, including increased levels of anxiety or depression, have been documented as being linked to coping through catastrophizing [30, 34]. A study by Rice et al. [35] reported that RA patients with specific dispositional affect patterns, identified through cluster analysis, may encounter heightened negative outcomes, including significant mood impairment, pain anxiety sensitivity, and pain catastrophizing [35]. Trait neuroticism was also recognised as a factor responsible for engaging in catastrophizing behaviours in response to pain [36]. Moreover, pain catastrophizing was hypothesised to be a possible link between neuroticism and pain [37]. This influence of psychological traits on pain catastrophizing is possibly reciprocal, as some results suggest that patients engaging in catastrophizing are susceptible to psychiatric comorbidities [38]. Religion may also exert an influence, as a collective analysis reported that spiritual well-being and pain intensity jointly explained 68% of the total variance in pain catastrophizing [39]. While caution is advised due to a methodology involving pooling of RA and axSpA cohorts, the results offer noteworthy insights and were therefore included in the present review.

Catastrophizing was also investigated from a sociological standpoint. Some authors found that a lack of support from family and friends was a key factor in predicting catastrophizing [29]. Additionally, there is evidence emphasising the importance of spouse relations [40]. It has been observed that individuals reporting higher satisfaction with their spouse's responses were less prone to experiencing enhanced negative emotions resulting from catastrophizing. The association between pain and catastrophizing diminished as satisfaction with spouse responses increased. Negative emotions were correlated with an increase in catastrophizing, but this link was statistically significant only when individuals reported decreased satisfaction with their spouse's responses. Previous experiences of childhood maltreatment were linked to higher odds of pain catastrophizing [41]. Importantly, whether the disease was considered active did not alter the connection between childhood maltreatment and pain catastrophizing. This association retained statistical significance even after adjusting for diverse factors, such as sociodemographic elements and symptoms of depression and anxiety.

Several analyses have highlighted the potential implications of higher pain catastrophizing on the disease course and patient-related outcomes. Most notably, it is consistently linked to a heightened perceived pain experience [42, 43]. A study by Lefebvre et al. [44] suggests that catastrophizing plays a crucial role in shaping how individuals remember their previous pain experiences [44]. Clinically, these findings imply that patients with higher catastrophizing scores may have a more precise recollection of their pain. While a connection between pain and catastrophizing exists, the impact of catastrophizing on physical limitations becomes more pronounced when acceptance levels are low [45]. In obese RA patients, higher PCS scores were associated with increased pain, reduced physical function, and a greater tendency to overeat [46]. Quality-of-life deterioration was consistently found to be associated with catastrophizing in various analyses [27, 43, 47, 48]. Berthelot et al. [49] reported significantly elevated Routine Assessment of Patient Index Data 3 (RAPID3) questionnaire scores in catastrophizers [49]. From a psychological standpoint, pain catastrophizing has emerged as a noteworthy mediator in the relationship between pain intensity, daily affect, and depressive symptoms [50]. Changes in daily pain have been associated with variations in pain catastrophizing, with increased catastrophizing leading to intensified emotional distress and depressive symptoms. Depression outcomes may also worsen within a 6-month follow-up period for patients engaging in catastrophizing [42]. Edwards et al. [32] reported that catastrophizing in RA patients was linked to higher psychological distress, increased pain intensity, and greater physical dysfunction, even after accounting for disease-related factors [32]. Interestingly, the magnitude of these associations was stronger among patients with lower levels of education or social functioning. Covic et al. [51] highlighted that pain catastrophizing shows a stronger connection to heightened pain and depression in RA compared to praying and hoping [51].

In certain studies, greater pain catastrophizing has been associated with a reduced likelihood of achieving remission [52, 53]. However, in another analysis, there was no independent association between higher levels of pain catastrophizing and the achievement of low disease activity [54]. Furthermore, pain catastrophizing has been identified as independent of inflammatory parameters [52, 55], implying its potential role in disease activity assessed with routine measures, even in the absence of evident inflammatory activity [53]. A clustering algorithm performed by Lee et al. [56] found that 47.3% of the analysed RA cohort experienced moderate-to-high levels of pain, fatigue, and sleep problems [56]. While most of these patients had low inflammatory markers, their high catastrophizing levels suggested a chronic widespread pain syndrome. Additionally, a discrepancy between patient global assessment (PGA) and the physician's assessment could be attributed to higher catastrophizing [57], but its role in the substantial discrepancy between the number of tender and swollen joints was not proven [58].

There is evidence suggesting that pain catastrophizing may decrease after initiating Disease Modifying Anti-Rheumatic Drug (DMARD) treatment [59–61]. This implies that efficiently managing disease activity, especially in relation to joint tenderness, can be beneficial in reducing pain catastrophizing. Additionally, psychological interventions and integrative medicine approaches have been shown to have a beneficial impact on pain catastrophizing in RA patient cohorts [62–64]. In a study by Davis et al. [65], mindfulness was highlighted for enhancing individuals' capacity to reduce catastrophizing when facing periods of elevated daily pain [65].

### Psoriatic arthritis

The initial search yielded nine potentially eligible articles. After removing duplicates and abstract and title screening, five articles remained. Subsequent full-text analysis confirmed that all five identified articles met the criteria for inclusion in the current review.

Limited information exists regarding the prevalence of pain catastrophizing in individuals with PsA. In a study involving 394 individuals with PsA, the mean simplified PCS score was 2.06, significantly better than in axSpA but not statistically different from patients with RA. The percentage of high pain catastrophizers (score ≥ 4) was 12.7%, with no statistically significant differences between RA and axSpA. Individuals with higher PCS scores tended to be younger and reported experiencing greater global pain, worse physical functioning, and impaired emotional well-being. Additionally, they had significantly lower levels of education. Higher pain catastrophizing scores were linked to significantly lower Health-Related Quality of Life (HRQoL) [27]. A further exploration of PCS in PsA was conducted in a study comparing PsA and a healthy control group, revealing a significant difference between them (16.95 vs. 3.75) [66].

In study by Currado et al. [67], the impact of pain catastrophizing was investigated in a cohort of 135 PsA patients, with 29.6% having concomitant fibromyalgia. Participants with a Disease Activity in Psoriatic Arthritis (DAPSA) score greater than 14 (the cut-off score between low and moderate activity) demonstrated notably higher scores related to pain catastrophizing. The mean PCS score in the DAPSA < 14 group was 7.5, whereas in the DAPSA ≥ 14 group, it was significantly greater at 29. Adjusted multivariable linear regression demonstrated a significant association between PCS and higher DAPSA scores. However, the presence of concomitant fibromyalgia raised concerns about the generalisability of the results. Interestingly, in another analysis, PCS was not significantly associated with low disease activity by DAPSA in regression models, but a low p-value (*p* = 0.11) may suggest some tendency that requires further study in larger cohorts [54].

In a randomised controlled trial by Vela et al. [68], the impact of cannabinoid use on pain and related factors, including pain catastrophizing, was investigated in patients with PsA. However, the study found that CBD did not result in clinically or statistically significant effects on pain intensity. Moreover, no statistically significant effects were noted for sleep quality, depression, anxiety, or pain catastrophizing scores [68].

### Axial spondyloarthritis

The initial search yielded 45 potentially eligible articles. After eliminating duplicates and screening of abstracts and titles, 11 articles remained. Subsequent full-text screening confirmed that all 11 identified articles met the criteria for inclusion in this review.

The mean overall PCS score in a Turkish axSpA cohort was 23.5 [69]. In a comparative analysis of PCS in major rheumatic musculoskeletal disorders, the simplified PCS in axSpA was 2.27, significantly higher than in RA and PsA [54]. Another analysis reported a median PCS score of 15 [70]. Available studies allow speculation regarding specific catastrophizer phenotypes in axSpA. In the Turkish cohort, the total PCS score weakly correlated with body mass index (BMI), pain, HRQoL, as well as fear and avoidance behaviours. Notably, BMI exhibited low and negative correlation with the total PCS score [69]. Another analysis revealed that younger patients with more physical issues, lower perceived general health, greater emotional problems, and lower levels of education were prone to experiencing heightened pain catastrophizing [27]. Additionally, one study underscored a possible negative link between spiritual well-being and PCS [39].

Other studies have indicated a correlation between PCS and elevated composite disease activity scores, such as Bath Ankylosing Spondylitis Disease Activity Index (BASDAI), emphasising the impact of maladaptive cognitive pain perception on achieving low disease activity and remission [54, 67]. Despite effective treatment of inflammation, individuals with possible central sensitisation may retain high disease activity scores. Moreover, a stronger perceived control over treatment was correlated with lower levels of catastrophizing thoughts, suggesting a potential link between perceived control and the degree of central sensitisation in these patients [70]. Higher pain catastrophizing was also associated with impaired HRQoL [27].

There is limited evidence regarding therapeutic approaches to catastrophizing in axSpA. One analysis aimed to create and assess an online pain management program for individuals with ankylosing spondylitis, integrating elements of mindfulness exercises, and cognitive–behavioural therapy (CBT). The program successfully decreased maladaptive thoughts and pain catastrophizing, and also promoted a more positive outlook on living with ankylosing spondylitis. However, there was a slight increase in the helplessness subscale of the PCS at the 10 week follow-up, suggesting a continued need for psychological support in managing ankylosing spondylitis. Despite these psychological benefits, the overall program did not effectively reduce pain intensity [71].

### Systemic sclerosis

The initial search identified five potentially eligible articles. After eliminating duplicates and screening of abstracts and titles, three articles remained. Subsequent full-text screening confirmed that all three articles were eligible for inclusion in the current review. Additionally, one more article was identified through reference tracking. Consequently, a total of four eligible articles were included in the current review.

Perrot et al. [30] conducted a study examining pain, its impact, and coping strategies in SSc and RA patients. The study included 82 SSc participants reporting a mean PCS score of 16.3. The observed disparity between SSc and RA was considered insignificant. The findings also indicated a correlation between depression, anxiety, and higher pain catastrophizing scores in individuals with SSc. In another study, individuals with SSc and lower levels of education were identified as particularly susceptible to engaging in pain-related catastrophizing [72].

Raynaud's phenomenon is a core symptom of SSc. An analysis found that the self-reported severity evaluation of SSc Raynaud's phenomenon is associated with various factors, including pain and catastrophizing [73]. Additionally, another study analysed the link between coping strategies and patient-perceived disability, patient global assessment, and physician global assessment in individuals with symptoms of Raynaud's syndrome. Maladaptive coping strategies, especially catastrophizing, were associated with elevated perceived disability and patient global assessment, even following adjustments for patient demographics, clinical phenotype, and disease severity evaluated by physician global assessment. These findings suggest that coping strategies, especially catastrophizing, may influence composite measures of disease severity, including the American College of Rheumatology Composite Index in Systemic Sclerosis (ACR CRISS) [74].

### Systemic lupus erythematosus

The initial search yielded 41 potentially eligible articles. After removal of duplicates and screening of titles and abstracts, six articles. Subsequent full-text screening confirmed that all six identified articles were eligible for inclusion in the current review.

Unfortunately, there are limited data on the prevalence of pain catastrophizing in the adult SLE population. Nevertheless, some paediatric studies have explored childhood-onset lupus. In one cohort with a mean age of 16.1, the average PCS score was 18.3, and 58% of participants reported a score equal to or above 15 [75]. Another prospective paediatric cohort with a mean age of 16.2 had a baseline mean PCS score of 17.9, which decreased to 15.4 after 6 months, although this change was not statistically significant. Medication regimens remained generally stable between visits. This emphasises that PCS may be a dynamic trait influenced by various biopsychosocial factors [76]. However, it is crucial to note that external validation for adult SLE patients might be limited based on the provided references.

Efforts have been made to recognise features predicting catastrophizing in SLE. In one study, a regression analysis indicated that kinesiophobia, depression, body awareness, and BMI were associated with increased pain catastrophizing [77]. Another analysis found that factors associated with catastrophizing included the total number of previous SLE medications, pain experienced in the last 7 days, mental HRQoL, and Systemic Lupus Activity Questionnaire score [78].

A Pain Coping Skills Training (PCST) program utilising the internet-based painTRAINER was evaluated in the SLE population. Pain catastrophizing and diverse outcomes, such as Patient-Reported Outcomes Measurement Information System (PROMIS) pain interference, sleep disruption, fatigue, anxiety, and depression, exhibited notable enhancements among painTRAINER users compared to the control group, with the most significant progress seen in pain catastrophizing [79].

### Sjögren’s syndrome

The initial search identified four articles that were potentially eligible. After removing duplicates, three articles remained following abstract and title screening. Subsequent examination of the full texts identified two articles that were deemed eligible for inclusion in the current review.

In one analysis, the mean PCS scores were 12.71 in seropositive and 13.16 in seronegative SS, showing no statistically significant difference [80]. Another comparative study including patients with fibromyalgia, RA, axSpA, and SS reported a mean catastrophizing score (CSQ) of 6.78 in patients with SS in a sample of 23 participants. Patients with fibromyalgia, rheumatoid arthritis, and spondyloarthritis had scores of 8.1, 6.34, and 7.30, respectively. Unfortunately, statistical tests for significant differences between the groups were not conducted [81].

In a study of the association of PCS in Sjögren’s syndrome with other factors, the most robust correlation was found between pain catastrophizing and pain anxiety. Linear regression analysis showed that catastrophizing was a stronger predictor of pain severity than age, fatigue, depression, or anxiety in both seropositive and seronegative primary SS patients. In a multivariate model derived through backward selection, four variables (pain catastrophizing, fibromyalgia status, serological status, and belief in severe consequences of illness) were responsible for 55% of the variance in pain severity. The study concluded that targeting pain catastrophizing and negative illness appraisal with behavioural interventions could be beneficial for individuals with primary SS [80]

### Juvenile idiopathic arthritis

The initial search identified 23 potentially eligible articles. After eliminating duplicates, 11 articles remained following abstract and title screening. Subsequent examination of the full texts identified eight articles that fulfilled the criteria for inclusion in the present review. This number was supplemented by two additional articles identified through reference tracking and one through citation tracking. Consequently, a total of 11 eligible articles were incorporated into this review.

The mean PCS score was 13.8 for a JIA cohort without concomitant fibromyalgia, consisting of 114 patients. In contrast, the JIA subgroup diagnosed with concomitant juvenile fibromyalgia exhibited significantly higher PCS scores at 28.2, and this group showed more pronounced functional impairment [82]. In an investigation of pain hypersensitivity within the JIA cohort, the median total score for the paediatric PCS was identified as 8 (with an interquartile range of 3–16) out of a possible 64 points [83].

A link between pain catastrophizing and the intensity of clinical and experimental pain has been hypothesised [84]. One study supports a model suggesting the significant influence of pain catastrophizing on pain experiences in JIA patients [85]. The results propose that targeting behavioural interventions at a specific subgroup of children, where pain experiences appear inconsistent with disease activity, could be an effective approach. Another analysis conducted among patients treated with TNF inhibitors indicated that individuals reporting high pain levels typically employed the pain-coping strategy of catastrophizing more frequently compared to pain-free children receiving the same treatment. These findings suggest that while a significant proportion of children respond well to anti-TNF treatment in terms of disease activity and pain, there remains a subgroup experiencing pain despite being in remission with biological agents [86]. JIA patients experiencing orofacial pain demonstrated a heightened level of catastrophizing compared to their healthy counterparts [87].

A study by Kyvsgaard et al. [88] reported that among children experiencing methotrexate-induced nausea, as determined by either the Methotrexate Intolerance Severity Score (MISS) or a nausea diary, there was a higher frequency of utilising the coping strategy of catastrophizing compared to other children. These psychological factors could contribute to the mechanism underlying individual differences in the severity of MTX-induced nausea in children with JIA [88].

Some evidence exists that, upon examining the potential impact of pain-coping strategies on the pain response, observations indicated that both children and parents who tended to employ the coping strategy of catastrophizing in response to experimental pain generally reported higher pain intensity, reduced pain tolerance, and increased discomfort related to pain [89]. Also, one interesting study suggested the hypothesis that parents with more negative, catastrophic thoughts about their child's pain were less likely to promote treatment adherence [90].

One study aimed to assess the practicability and initial effectiveness of a CBT intervention for children with JIA and their parents [91]. Despite pain management being the primary focus, there was no observed reduction in pain following the intervention. However, preliminary analysis indicated that, despite an increase in disease severity, the intervention group experienced improvements in QoL, a decrease in pain catastrophizing, and e nhanced adaptive pain cognitions (beliefs in controlling pain and self-efficacy). The study underscores the importance of considering disease status when evaluating the efficacy of psychological interventions in paediatric arthritis. Its limitations included a small study population, a low response rate, and a short period of observation.

### Osteoarthritis

The initial search yielded 520 articles that could potentially be included. After eliminating duplicates, 179 articles remained following abstract and title screening. Further analysis of the full texts identified 75 articles that were considered suitable for inclusion in the present review. This number was augmented by six additional articles identified through citation tracking. One more article was identified on additional screening as of submission date. Consequently, a total of 82 eligible articles were incorporated into the present review.

The prevalence of pain catastrophizing may vary due to the diverse anatomical locations of different types of OA. This is because various joint pains and disabilities may impact individuals' function and overall well-being in different ways. Unfortunately, this variability poses a challenge in obtaining specific data on this matter. In cohorts with combined types of OA, the mean PCS scores ranged from 14.4 to 19.1 [92, 93]. In cases of severe hip OA, the median PCS value was identified as 26 [94]. For knee OA, mean PCS scores ranged from 15.2 to 23.0 [95–97]. Our literature search, which focused on conservatively treated OA, did not yield direct comparisons across different localisations of OA-affected joints. Additionally, there is a lack of data comparing the PCS scores of patients with OA with healthy controls or individuals with other rheumatic disorders.

There are conflicting reports on the association of gender with pain catastrophizing in OA cohorts: one study concluded that women are more prone to catastrophizing than men [98], while another found no such differences [99]. One analysis reported that pain catastrophizing is notably higher in morbidly obese OA patients compared to their overweight and obese counterparts [100]. High levels of pain catastrophizing were correlated with more intense and unpleasant pain, increased binge eating, lower self-efficacy in controlling eating, and reduced weight-related QoL. In a knee OA cohort in Nigeria, positive correlations were also observed between increased BMI and pain catastrophizing [101]. However, another study showed no links between pain catastrophizing and BMI, nor age and radiographic severity [102]. Regarding socioeconomic factors, lower levels of education were independently linked to the co-occurrence of pain catastrophizing and fear of movement in OA [103]. Catastrophic thinking was found to be prevalent in patients with low radiographic severity, particularly those experiencing high pain intensity [104]. Furthermore, in one analysis, catastrophizing had a more robust association with pain in specific participants who were younger, overweight, had more comorbidities, or reported poorer sleep quality [105].

Racial disparities in PCS were investigated in various analyses. In a study by Nemati et al. [106] focusing on PCS domains, structural equation modelling revealed that among Hispanics, pain severity correlated with rumination, magnification, and helplessness, while lower physical function scores were associated with greater magnification and helplessness [106]. Among non-Hispanic Whites, pain severity was linked to rumination and helplessness. In a 2-year follow-up study involving 187 adults with knee OA, Fullwood [107] reported that pain catastrophizing mediated the relationship between ethnicity and pain, disability, and physical function [107].

In contrast with individuals of non-Hispanic White descent, non-Hispanic Black participants reported higher levels of pain and disability coupled with lower physical function [108]. This influenced by heightened levels of catastrophizing among non-Hispanic Black individuals (1.7 vs. 0.9, *p* < 0.01, on a 0–6 CSQ scale) [108]. Similarly, in another study, Japanese participants exhibited higher pain catastrophizing scores compared to their Australian counterparts [109].

Several factors have been identified as contributors to heightened catastrophizing in OA. Psychological elements, including increased ambivalence over emotional expression and reduced self-efficacy for pain communication, may increase the likelihood of pain catastrophizing [110]. Additionally, there is evidence indicating that older perceived age is associated with increased pain catastrophizing while being also associated with trait resilience and positive affect [111]. Furthermore, a correlation between sleep disturbances and increased pain catastrophizing [112].

Pain catastrophizing is a dynamic trait that may vary over time in individuals with OA, particularly intensifying during periods of heightened pain [113]. Information regarding the locations and patterns of pain is also crucial: higher levels of pain catastrophizing were observed in cases of a diffuse type of knee pain [114] Furthermore, in some individuals with knee OA, there are reports of perceived swelling in the knee without clear evidence, also potentially indicating elevated levels of pain catastrophizing [115].

Neurobiological studies shed light on the pathophysiology of catastrophizing in OA. The mid-anterior cingulate cortex (mACC), which is crucial in pain sensation, has low γ-aminobutyric acid (GABA) levels in OA which correlates with greater pain, suggesting a role in prefrontal disinhibition. A study by El-Najjar et al. [116] using magnetic resonance spectroscopy explored mACC GABA levels in patients with chronic knee OA pain, revealing a negative correlation between mACC GABA and the PCS [116]. The myo-inositol:Glx ratio was also significantly correlated with PCS. GABA and myo-inositol:Glx were proposed as potential biomarkers for chronic knee OA pain. Addressing racial disparities in brain structure and function, Terry's study [117] reported that greater pain catastrophizing was associated with a thinner primary somatosensory cortex in non-Hispanic Whites but not in non-Hispanic Blacks, suggesting distinct effects on the emotional–motivational aspect of the pain system for non-Hispanic Blacks [117]. Another study by Terry [118] investigated the impact of ethnicity on the links between pain catastrophizing, clinical pain, and resting-state functional connectivity in specific brain areas—namely, the anterior cingulate cortex (ACC), dorsolateral prefrontal cortex (dlPFC), insula, and primary somatosensory cortex (S1) [118]. In 136 adults with knee OA, ethnicity influenced the mediation effects of catastrophizing on the relationship between clinical pain and resting-state functional connectivity in the ACC, dlPFC, insula, and S1, suggesting distinct relationships for non-Hispanic Black people and non-Hispanic Whites.

In the existing literature on OA, heightened levels of catastrophizing have been consistently linked to lower pain thresholds, reduced pain tolerance, higher laboratory pain ratings, and increased clinical pain levels [99]. In the same analysis, catastrophizing appeared to be associated with subjective pain reports rather than the nociceptive flexion reflex threshold, indicating a connection with an intensified pain experience rather than the modulation of spinal gating mechanisms. Some authors have proposed that catastrophizing acts as a mediator in the relationship between pain-related unpleasantness and suffering [119]. Numerous analyses have demonstrated that an elevated PCS score correlates with higher pain levels in OA [96, 120–122]. In certain analyses, this association remained independent of other factors such as depression, anxiety, stress, and affect [123].

In one analysis, pain catastrophizing was integrated into a predictive model designed to forecast pain relief during conservative treatment, demonstrating moderate effectiveness [124]. Several longitudinal studies have indicated that pain catastrophizing diminishes the likelihood of achieving favourable pain outcomes [125, 126]. Additionally, catastrophizing was identified as a mediator in the association between gender and OA pain-related results. Notably, even after accounting for depression, catastrophizing persisted in mediating the relationship between gender and pain, suggesting distinct effects not solely explained by depression alone [98]. Conversely, some reports suggest that psychological traits, including catastrophizing, do not act as mediators in the phenotypic differences in pain sensitivity observed among individuals with advanced knee OA [127]. Chronic pain in knee OA often exhibits similarities to neuropathic pain, with psychological factors such as high pain catastrophizing playing a role. A study by Tanaka et al. [128] identified a significant association between elevated pain catastrophizing and the existence of a neuropathic pain aspect in symptomatic knee OA, as assessed by the painDETECT questionnaire [128]. The PCS was also established as a significant predictor of higher Western Ontario and McMaster Universities Arthritis Index (WOMAC) levels [95, 129–131].

Catastrophizing proves to be valuable in distinguishing between older adults with OA experiencing chronic pain, with and without depressive symptomatology [132]. Additionally, it has been noted that women who engage in catastrophizing are less prone than men to report a negative mood [99, 133]. An association between catastrophizing and empathy-related responses in knee OA patients has also been observed [134]. Furthermore, pain catastrophizing emerged as a significant mediator in the correlation between dispositional optimism and temporal summation of heat pain, even after adjusting for confounding factors. Psychologically resilient individuals with high dispositional optimism may exhibit fewer maladaptive pain responses [135]. Individuals with OA who practiced catastrophizing in the morning reported elevated negative mood and reduced positive mood in the evening, irrespective of fluctuations in pain throughout the day [113, 133]. The impact of pain catastrophizing in OA patients was also examined within the context of relationships in several analyses. Among patients, greater self-efficacy was associated with lower pain measures, including catastrophizing, while increased holding back correlated with higher psychological disability and catastrophizing. Partner holding back was also linked to elevated levels of catastrophizing [136]. Another analysis found that when patients reported higher morning catastrophizing, their spouses encountered increased negative affect throughout the day. Moreover, punishing responses from spouses predicted increased pain catastrophizing in patients the following morning, independent of patient pain and negative affect [137]. Another analysis revealed that both trait and state pain catastrophizing influenced the connection between daily partner support and pain intensity. During days characterised by low partner support, individuals with high levels of catastrophizing reported greater pain intensity compared to those with low levels of catastrophizing. However, when partner support was higher, pain intensity did not vary between individuals with high and low levels of catastrophizing. This suggests that partner support may play a vital role in mitigating pain intensity, particularly for individuals with higher levels of pain catastrophizing [138].

A significant association between sleep disturbances and heightened pain catastrophizing has been demonstrated which, in turn, correlates with increased severity of OA symptoms or depressive symptoms [93, 139]. Conversely, pain catastrophizing has also been identified as a predictor of sleep problems, surpassing the influence of depression and pain [140]. Catastrophizing plays a moderating role in the relationship between sleep efficiency and central sensitisation. More precisely, individuals with low sleep efficiency and high catastrophizing scores had greater levels of central sensitisation. These findings provide initial evidence supporting a synergistic impact of catastrophizing and sleep, leading to increased pain sensitivity in patients with OA [141]. One study indicated that improvements in short-term sleep were not statistically associated with reductions in depressive symptoms or pain catastrophizing [142]. However, in another sample of knee OA patients with coexisting insomnia, interventions targeting sleep led to a decrease in all three measures of pain catastrophizing. These reductions were evident after 8 weeks of treatment and remained consistent at the 6-month follow-up [143].

Pain catastrophizing has been established as a significant factor negatively associated with HRQoL [144, 145]. An analysis demonstrated this influence, highlighting its significance regardless of disease severity [102]. The importance of considering pain catastrophizing in these assessments was underscored by the results of an analysis revealing that it exhibited either the strongest or second strongest impact on QoL, surpassed only by pain intensity [94].

There is evidence suggesting that pain catastrophizing is a well-established factor associated with disability and reduced physical function [92, 96, 97, 146]. In some sources, this association is significant and independent, even after adjusting for pain levels [147]. These findings may be explained by the hypothesis that heightened sensitivity to physical activity might be predicted by pain catastrophizing [148]. A relationship between higher morning pain catastrophizing and increased sedentary time and reduced physical activity on the same day has been reported [149]. Additionally, the location of arthritis and its severity as shown in radiographs were found to not be associated with either physical function or pain, suggesting that limitations resulting from OA are more strongly linked to personal and psychological factors, particularly ineffective cognitive coping mechanisms such as catastrophizing [150]. Another analysis also suggested that reduced capability is linked to cognitive biases about pain, specifically catastrophic ones, rather than the Kellgren–Lawrence grade of radiographic OA [151]. Some of these associations may be elucidated by the link between pain catastrophizing and muscle weakness [152]. Another analysis identified a connection between catastrophizing and isometric knee strength; however, they were not independent predictors of isokinetic strength, knee pain, or physical performance [153].

Individuals with knee OA may encounter instability and occasional buckling. Postural stability has been identified as negatively associated with pain catastrophizing. Also, elevated levels of pain-related fear and catastrophizing were connected to an avoidance response, which could potentially lead to chronic disability [154]. However, conflicting results are presented in some studies: for example, suggesting no relationship between pain catastrophizing to fear of falling, the number of falls, and static balance [155, 156]. Certain studies highlight the potential significance of catastrophizing in gait velocity and walking behaviour, emphasising its role as a possible therapeutic factor [147, 157]. However, conflicting results have been presented in other analyses, suggesting that depressive symptoms, rather than catastrophizing or baseline living situation, are associated with a significant decline in daily walking [158]. In an analysis involving 151 participants, individuals with pain catastrophizing exhibited a significant decrease in stair climbing ability, even after adjusting for covariates and a sensitivity analysis [159]. However, this association was not observed for the ability to stand from a seated position and walk.

The Lequesne Index (LI) is a tool that evaluates OA severity in the hips and knees, considering pain, walking distance, and daily living impact. A higher score indicates more severe OA-related impairment. In a study focusing on knee OA, a weak correlation was found between LI and radiographic severity, with no correlation between PCS and the radiographic scale. However, a moderate association was observed between PCS and the LI. Interestingly, patients seen by rheumatologists had higher PCS scores than those seen by general practitioners, despite similar radiographic and LI scores [160].

Research indicates that catastrophizing in OA may be a factor that can be modified. One proposed approach is to focus on enhancing pain-coping skills. Participating in web-based training specifically targeting pain-coping skills was found to result in a decrease in the utilisation of maladaptive behavioural strategies such as catastrophizing [161]. In a cohort of individuals with both obesity and overweight suffering from OA, the impact was more significant when pain-coping skills’ training was combined with behavioural strategies for weight control. Consequently, there was a notable reduction in pain catastrophizing compared to groups receiving standard care. Additionally, they exhibited significantly improved outcomes in areas such as pain, physical disability, stiffness, activity, weight self-efficacy, and weight [162]. However, despite a decrease in pain catastrophizing, some studies suggest that pain-coping skills may not effectively reduce pain severity in African Americans with OA [163]. In a study involving 300 veteran patients with OA, a 12-month phone-based behavioural protocol did not lead to a reduction in catastrophizing compared to the control group halfway through the treatment [164].

Moreover, a hypothesised connection between catastrophizing and self-efficacy has been explored. A study by Shelby in 2008 [165] reported that pain catastrophizing heightened pain and disability by diminishing self-efficacy, even after adjusting for demographic and clinical factors [165]. The reduction of pain catastrophizing has been associated with improved self-efficacy which, when combined with increased social support, can lead to an enhancement in health status [166]. Additionally, some suggest that prioritising the improvement of self-efficacy is more crucial than directly addressing catastrophizing [167]. The study recommended interventions that target both self-efficacy and catastrophizing for a more significant impact on physical functioning compared to treatments with a single focus.

A study found that both periodised circuit training and conventional strength training significantly decreased pain catastrophizing [168]. In relation to pain catastrophizing, it has been suggested that incorporating pain neuroscience education followed by Pilates exercises compared to Pilates exercises alone can result in statistically significant improvement in PCS [169]. The addition of action observation therapy to an exercise program for pain and related measures was also explored. While both the treatment and control groups exhibited significant improvement in all outcomes, including catastrophizing, there was no notable difference between them [170]. Incorporating pain neuroscience education alongside conventional physiotherapy exercises was discovered to lead to a greater reduction in pain catastrophizing compared to the conventional physiotherapy alone in patients with knee osteoarthritis [171]. An investigation into the impact of exercise on the prefrontal cortex in knee OA explored with functional near-infrared spectroscopy indicated a reduction in dorsolateral prefrontal cortex (DLPFC) activity during painful stimuli after exercise. Changes in DLPFC activation were correlated with improvements in pain perception and pain catastrophizing scores [172]. Additionally, catastrophizing itself can influence the outcomes of physical therapy. Patients with low levels of pain catastrophizing tended to show more substantial improvement with physical therapy at weeks 2 and 6. A multivariate logistic regression analysis showed that the baseline PCS score was a significant predictor for both pain and function at week 6. In contrast, the baseline depression score did not independently predict a clinically poor outcome [173]. No clinically or statistically significant effects of CBD on pain intensity were observed in patients with hand OA and PsA when compared to placebo. Furthermore, there were no statistically significant effects on sleep quality, depression, anxiety, or pain catastrophizing scores [68].

## Conclusion

The current scientific evidence highlights the importance of assessing pain catastrophizing in the management of rheumatic musculoskeletal disorders. This review clarifies and brings attention to this phenomenon, which appears to be often overlooked in patients attending rheumatology clinics. This article highlights a number of factors responsible for underdiagnosis of pain catastrophizing. Their recognition could enhance detection and, consequently, facilitation of targeted therapeutic interventions. Pain catastrophizing has diverse effects on various aspects of overall health, as demonstrated in this review, emphasising its substantial significance in patient care. Increased awareness and understanding of pain catastrophizing within rheumatic musculoskeletal disorders could lead to a more effective diagnostic approach and tailored treatment strategy, ultimately improving patient healthcare and health outcomes.

Our understanding of pain catastrophizing in rheumatic disorders is nascent and there is a significant knowledge gap necessitating further exploration. Understanding the genesis of pain catastrophizing, particularly from comprehensive biopsychosocial perspectives across rheumatic disorders, appears to be crucial. Future research should delve into these mechanisms to advance our understanding of pain perception and management in rheumatology. Survey studies among rheumatologists and patients could help to elucidate knowledge gaps, while expansive cohort studies could reveal the prevalence of pain catastrophizing across various rheumatic disorders and compare it with other musculoskeletal conditions. Identifying core variables and predisposing factors associated with pain catastrophizing could allow the construction of a predictive model for clinical probability scoring to facilitate targeted interventions. Exploring the influence of pain catastrophizing on rheumatic disorders and assessing treatment approaches targeting pain catastrophizing are crucial. A holistic approach is vital for advancing rheumatology, enhancing patient care strategies, and improving the quality of life for individuals with rheumatic musculoskeletal disorders.

Regarding our study's strengths, we emphasise the methodological rigor employed during the search and writing processes. We conducted a comprehensive search across major biomedical literature databases, including open-access articles indexed in DOAJ, and supplemented this with citation and reference screening. Our study is the first narrative review consolidating existing knowledge on pain catastrophizing within major rheumatic disorders, making a seminal contribution to this important topic. However, limitations include the single researcher conducting the search, potentially introducing bias, and the exclusion of studies related to surgically treated OA and orthopaedic surgery qualifications, which could limit the scope of our analysis.

In conclusion, the significance of pain catastrophizing in rheumatic disorders deserves heightened attention due to its potential clinical implications, which may contribute to overall health deterioration. However, it is essential to acknowledge that certain knowledge gaps persist, and further high-quality research is imperative to bridge these gaps and advance our understanding of the complex interplay between pain catastrophizing and rheumatic disorders.

### Supplementary Information

Below is the link to the electronic supplementary material.

Supplementary file1 (DOCX 20 KB)

## Data Availability

The data underlying this study is available upon reasonable request.

## References

[1] Ziade N, El Khoury B, Zoghbi M (2020). (2020) Prevalence and pattern of comorbidities in chronic rheumatic and musculoskeletal diseases: the COMORD study. Sci Rep.

[2] Liu S, Wang B, Fan S (2022). Original research: global burden of musculoskeletal disorders and attributable factors in 204 countries and territories: a secondary analysis of the global burden of disease 2019 study. BMJ Open.

[3] Gill TK, Mittinty MM, March LM (2023). Global, regional, and national burden of other musculoskeletal disorders, 1990–2020, and projections to 2050: a systematic analysis of the global burden of disease study 2021. Lancet Rheumatol.

[4] Hadi MA, McHugh GA, Closs SJ (2019). Impact of chronic pain on patients’ quality of life: a comparative mixed-methods study. J Patient Exp.

[5] Garrido-Cumbrera M, Hillmann O, Mahapatra R (2017). Improving the management of psoriatic arthritis and axial spondyloarthritis: roundtable discussions with healthcare professionals and patients. Rheumatol Ther.

[6] Pomarensky M, Macedo L, Carlesso LC (2022). Management of chronic musculoskeletal pain through a biopsychosocial lens. J Athl Train.

[7] Gibson E, Sabo MT (2018). Can pain catastrophizing be changed in surgical patients? A scoping review. Can J Surg.

[8] Miller MM, Meints SM, Hirsh AT (2018). Catastrophizing, pain, and functional outcomes for children with chronic pain: a meta-analytic review. Pain.

[9] Quartana PJ, Campbell CM, Edwards RR (2009). Pain catastrophizing: a critical review. Expert Rev Neurother.

[10] Petrini L, Arendt-Nielsen L (2020). Understanding pain catastrophizing: putting pieces together. Front Psychol.

[11] Ellis A (1962). Reason and emotion in psychotherapy.

[12] Beck A, Rush A, Shaw B, Emery G (1979). Cognitive therapy of depression.

[13] Rosenstiel AK, Keefe FJ (1983). The use of coping strategies in chronic low back pain patients: relationship to patient characteristics and current adjustment. Pain.

[14] Sullivan MJL, Bishop SR, Pivik J (1995). The pain catastrophizing scale: development and validation. Psychol Assess.

[15] Majumder MSM, Ahmed S, Shazzad N (2020). Translation, cross-cultural adaptation and validation of the pain catastrophizing scale (PCS) into Bengali in patients with chronic non-malignant musculoskeletal pain. Int J Rheum Dis.

[16] Fernandes L, Storheim K, Lochting I, Grotle M (2012). Cross-cultural adaptation and validation of the Norwegian pain catastrophizing scale in patients with low back pain. BMC Musculoskelet Disord.

[17] Marttinen MK, Santavirta N, Kauppi MJ (2018). Validation of the pain coping questionnaire in Finnish. Eur J Pain.

[18] Lewis GN, Rice DA, McNair PJ, Kluger M (2015). Predictors of persistent pain after total knee arthroplasty: a systematic review and meta-analysis. Br J Anaesth.

[19] Vissers MM, Bussmann JB, Verhaar JAN (2012). Psychological factors affecting the outcome of total hip and knee arthroplasty: a systematic review. Semin Arthritis Rheum.

[20] Ashoorion V, Sadeghirad B, Wang L (2023). Predictors of persistent post-surgical pain following total knee arthroplasty: a systematic review and meta-analysis of observational studies. Pain Med.

[21] Burns LC, Ritvo SE, Ferguson MK (2015). Pain catastrophizing as a risk factor for chronic pain after total knee arthroplasty: a systematic review. J Pain Res.

[22] Gasparyan AY, Ayvazyan L, Blackmore H, Kitas GD (2011). Writing a narrative biomedical review: considerations for authors, peer reviewers, and editors. Rheumatol Int.

[23] Ferrari R (2015). Writing narrative style literature reviews. Med Writ.

[24] Baethge C, Goldbeck-Wood S, Mertens S (2019). SANRA—a scale for the quality assessment of narrative review articles. Res Integr Peer Rev.

[25] Green BN, Johnson CD, Adams A (2006). Writing narrative literature reviews for peer-reviewed journals: secrets of the trade. J Chiropr Med.

[26] Lee YC, Lu B, Edwards RR (2013). The role of sleep problems in central pain processing in rheumatoid arthritis. Arthritis Rheum.

[27] Wilk M, Łosińska K, Pripp AH (2022). Pain catastrophizing in rheumatoid arthritis, psoriatic arthritis and axial spondyloarthritis: biopsychosocial perspective and impact on health-related quality of life. Rheumatol Int.

[28] Jensen MP, Keefe FJ, Lefebvre JC (2003). One- and two-item measures of pain beliefs and coping strategies. Pain.

[29] Penhoat M, Saraux A, Le Goff B (2014). High pain catastrophizing scores in one-fourth of patients on biotherapy for spondylarthritis or rheumatoid arthritis. Joint Bone Spine.

[30] Perrot S, Dieudé P, Pérocheau D, Allanore Y (2013). Comparison of pain, pain burden, coping strategies, and attitudes between patients with systemic sclerosis and patients with rheumatoid arthritis: a cross-sectional study. Pain Med (United States).

[31] Watkins KW, Shifren K, Park DC, Morrell RW (1999). Age, pain, and coping with rheumatoid arthritis. Pain.

[32] Edwards RR, Giles J, Bingham CO (2010). Moderators of the negative effects of catastrophizing in arthritis. Pain Med.

[33] Hammer HB, Michelsen B, Sexton J (2021). Fatigue is cross-sectionally not associated with objective assessments of inflammation, but changes in fatigue are associated with changes of disease activity assessments during biologic treatment of patients with established rheumatoid arthritis. Clin Rheumatol.

[34] Ziarko M, Siemiatkowska K, Sieński M (2019). Mental health and rheumatoid arthritis: toward understanding the emotional status of people with chronic disease. Biomed Res Int.

[35] Rice DB, Mehta S, Pope JE (2016). Dispositional affect in unique subgroups of patients with rheumatoid arthritis. Pain Res Manag.

[36] Affleck G, Tennen H, Urrows S, Higgins P (1992). Neuroticism and the pain-mood relation in rheumatoid arthritis: insights from a prospective daily study. J Consult Clin Psychol.

[37] Gharavi Roudsari E, Mousavi Nasab SMH, Ghavidel-Parsa B (2022). Personality and pain intensity in rheumatoid arthritis patients: the mediating role of pain catastrophizing and cognitive emotion regulation strategies. Pers Individ Dif.

[38] Zyrianova Y, Kelly BD, Sheehan J (2011). The psychological impact of arthritis: the effects of illness perception and coping. Ir J Med Sci.

[39] Korkut S, Ülker T, Saatçi G (2023). The power of spiritual well-being: its relationship with pain intensity, pain management, and pain catastrophizing in individuals with chronic pain. Pain Manag Nurs.

[40] Holtzman S, DeLongis A (2007). One day at a time: the impact of daily satisfaction with spouse responses on pain, negative affect and catastrophizing among individuals with rheumatoid arthritis. Pain.

[41] MacDonald TM, Fisk JD, Bernstein CN (2021). The association between childhood maltreatment and pain catastrophizing in individuals with immune-mediated inflammatory diseases. J Psychosom Res.

[42] Keefe FJ, Brown GK, Wallston KA, Caldwell DS (1989). Coping with rheumatoid arthritis pain: catastrophizing as a maladaptive strategy. Pain.

[43] Hashimoto A, Sonohata M, Mawatari M (2020). The use of oral analgesics and pain self-efficacy are independent predictors of the quality of life of individuals with rheumatoid arthritis. Pain Res Manag.

[44] Lefebvre JC, Keefe FJ (2002). Memory for pain: the relationship of pain catastrophizing to the recall of daily rheumatoid arthritis pain. Clin J Pain.

[45] Costa J, Pinto-Gouveia J, Marôco J (2014). Pain related catastrophizing on physical limitation in rheumatoid arthritis patients. Is acceptance important?. Span J Psychol.

[46] Somers TJ, Wren AA, Blumenthal JA (2014). Pain, physical functioning, and overeating in obese rheumatoid arthritis patients: do thoughts about pain and eating matter?. J Clin Rheumatol.

[47] Piarulli A, Conversano C, Ciacchini R (2021). Catastrophisation, chronic pain and sexuality: a cross-sectional investigation in fibromyalgia and rheumatoid arthritis. Clin Exp Rheumatol.

[48] Larice S, Ghiggia A, Di Tella M (2020). Pain appraisal and quality of life in 108 outpatients with rheumatoid arthritis. Scand J Psychol.

[49] Berthelot JM, Bart G, Darrieutort-Lafitte C (2019). Pain catastrophising worsens RAPID3 in all rheumatologic conditions. Clin Exp Rheumatol.

[50] Sturgeon JA, Zautra AJ (2013). State and trait pain catastrophizing and emotional health in rheumatoid arthritis. Ann Behav Med.

[51] Covic T, Adamson B, Spencer D, Howe G (2003). A biopsychosocial model of pain and depression in rheumatoid arthritis: a 12-month longitudinal study. Rheumatology (Oxford).

[52] Hammer HB, Uhlig T, Kvien TK, Lampa J (2018). Pain catastrophizing, subjective outcomes, and inflammatory assessments including ultrasound: results from a longitudinal study of rheumatoid arthritis patients. Arthritis Care Res (Hoboken).

[53] Yoshida T, Hashimoto M, Horiguchi G (2021). Pain catastrophizing hinders disease activity score 28 – erythrocyte sedimentation rate remission of rheumatoid arthritis in patients with normal C-reactive protein levels. Int J Rheum Dis.

[54] Wilk M, Pripp AH, Korkosz M, Haugeberg G (2023). Exploring pain catastrophizing and its associations with low disease activity in rheumatic inflammatory disorders. Rheumatol Int.

[55] Abe T, Tamura M, Azuma N, Matsui K (2023). The role of pain catastrophizing in pain perception among patients with rheumatoid arthritis without clinical signs of inflammation. Musculoskelet Care.

[56] Lee YC, Frits ML, Iannaccone CK (2014). Subgrouping of rheumatoid arthritis patients based on pain, fatigue, inflammation and psychosocial factors. Arthritis Rheumatol.

[57] Hayashi K, Miki K, Shi K (2023). Discordance of global assessment between the patients and physicians predicts 9-year pain-related outcomes in rheumatoid arthritis patients. Front Med (Lausanne).

[58] Jansen N, ten Klooster PM, Vonkeman HE (2023). Further evaluation of inflammatory and non-inflammatory aspects of pain in rheumatoid arthritis patients. Rheumatol Adv Pract.

[59] Shim EJ, Song YW, Park SH (2017). Examining the relationship between pain catastrophizing and suicide risk in patients with rheumatic disease: the mediating role of depression, perceived social support, and perceived burdensomeness. Int J Behav Med.

[60] Salaffi F, Carotti M, Farah S (2022). Early response to JAK inhibitors on central sensitization and pain catastrophizing in patients with active rheumatoid arthritis. Inflammopharmacology.

[61] Cohen EM, Edwards RR, Bingham CO (2019). Pain and catastrophizing in patients with rheumatoid arthritis: an observational cohort study. J Clin Rheumatol.

[62] Zautra AJ, Davis MC, Reich JW (2008). Comparison of cognitive behavioral and mindfulness meditation interventions on adaptation to rheumatoid arthritis for patients with and without history of recurrent depression. J Consult Clin Psychol.

[63] Sinclair VG (2001). Predictors of pain catastrophizing in women with rheumatoid arthritis. Arch Psychiatr Nurs.

[64] Kwissa-Gajewska Z, Olesińska M, Tomkiewicz A (2014). Optimism, pain-coping strategies and pain intensity among women with rheumatoid arthritis. Reumatologia.

[65] Davis MC, Zautra AJ, Wolf LD (2015). Mindfulness and cognitive-behavioral interventions for chronic pain: differential effects on daily pain reactivity and stress reactivity. J Consult Clin Psychol.

[66] Vela J, Dreyer L, Petersen KK (2023). Quantitative sensory testing, psychological profiles and clinical pain in patients with psoriatic arthritis and hand osteoarthritis experiencing pain of at least moderate intensity. Eur J Pain.

[67] Currado D, Biaggi A, Pilato A (2023). The negative impact of pain catastrophising on disease activity: analyses of data derived from patient-reported outcomes in psoriatic arthritis and axial spondyloarthritis. Clin Exp Rheumatol.

[68] Vela J, Dreyer L, Petersen KK (2022). Cannabidiol treatment in hand osteoarthritis and psoriatic arthritis: a randomized, double-blind, placebo-controlled trial. Pain.

[69] İlçin N, Gürpınar B, Bayraktar D (2016). Cross-cultural adaptation and validation of the Turkish version of the pain catastrophizing scale among patients with ankylosing spondylitis. J Phys Ther Sci.

[70] Kieskamp SC, Paap D, Carbo MJG (2021). Central sensitization, illness perception and obesity should be considered when interpreting disease activity in axial spondyloarthritis. Rheumatology (Oxford).

[71] Yu KC, Lo LY, Lin M (2021). A preliminary study of an online pain management programme for patients with ankylosing spondylitis. Couns Psychother Res.

[72] Edwards RR, Goble L, Kwan A (2006). Catastrophizing, pain, and social adjustment in scleroderma: relationships with educational level. Clin J Pain.

[73] Pauling JD, Reilly E, Smith T, Frech TM (2019). Factors influencing raynaud condition score diary outcomes in systemic sclerosis. J Rheumatol.

[74] DiRenzo DD, Smith TR, Frech TM (2021). Effect of coping strategies on patient and physician perceptions of disease severity and disability in systemic sclerosis. J Rheumatol.

[75] Jones JT, Cunningham N, Kashikar-Zuck S, Brunner HI (2016). Pain, fatigue and psychological impact on health-related quality of life in childhood-onset lupus. Arthritis Care Res (Hoboken).

[76] Donnelly C, Cunningham N, Jones JT (2018). Fatigue and depression predict reduced health-related quality of life in childhood-onset lupus. Lupus.

[77] Kinikli GI, Bal GA, Aydemir-Guloksuz EG, Kinikli G (2022). Predictors of pain catastrophizing in women with systemic lupus erythematosus. Rev Assoc Med Bras.

[78] Fischin J, Chehab G, Richter JG (2015). Factors associated with pain coping and catastrophising in patients with systemic lupus erythematosus: a cross-sectional study of the LuLa-cohort. Lupus Sci Med.

[79] Allen KD, Beauchamp T, Rini C (2021). Pilot study of an internet-based pain coping skills training program for patients with systemic lupus erythematosus. BMC Rheumatol.

[80] Segal BM, Pogatchnik B, Rhodus N (2014). Pain in primary Sjögren’s syndrome: the role of catastrophizing and negative illness perceptions. Scand J Rheumatol.

[81] Bucourt E, Martaillé V, Goupille P (2021). A comparative study of fibromyalgia, rheumatoid arthritis, spondyloarthritis, and Sjögren’s syndrome; impact of the disease on quality of life, psychological adjustment, and use of coping strategies. Pain Med.

[82] Tesher MS, Graham TB, Ting T (2022). Juvenile fibromyalgia in patients with juvenile idiopathic arthritis: utility of the pain and symptom assessment tool. Arthritis Care Res (Hoboken).

[83] Cornelissen L, Donado C, Kim J (2014). Pain hypersensitivity in juvenile idiopathic arthritis: a quantitative sensory testing study. Pediatr Rheumatol Online J.

[84] Thastum M, Zachariae R, Schøler M, Herlin T (1999). A Danish adaptation of the pain coping questionnaire for children: preliminary data concerning reliability and validity. Acta Paediatr.

[85] Thastum M, Herlin T, Zachariae R (2005). Relationship of pain-coping strategies and pain-specific beliefs to pain experience in children with juvenile idiopathic arthritis. Arthritis Rheum.

[86] Lomholt JJ, Thastum M, Herlin T (2013). Pain experience in children with juvenile idiopathic arthritis treated with anti-TNF agents compared to non-biologic standard treatment. Pediatr Rheumatol Online J.

[87] Dimitrijevic Carlsson A, Wahlund K, Kindgren E (2019). Orofacial pain in juvenile idiopathic arthritis is associated with stress as well as psychosocial and functional limitations. Pediatr Rheumatol Online J.

[88] Kyvsgaard N, Thastum M, Mikkelsen TS (2020). Coping strategies and anxiety in association with methotrexate-induced nausea in juvenile idiopathic arthritis. Rheumatol Int.

[89] Thastum M, Zachariae R, Schøler M (1997). Cold pressor pain: comparing responses of juvenile arthritis patients and their parents. Scand J Rheumatol.

[90] Brandelli YN, Chambers CT, Tutelman PR (2019). Parent pain cognitions and treatment adherence in juvenile idiopathic arthritis. J Pediatr Psychol.

[91] Lomholt JJ, Thastum M, Christensen AE (2015). Cognitive behavioral group intervention for pain and well-being in children with juvenile idiopathic arthritis: a study of feasibility and preliminary efficacy. Pediatr Rheumatol Online J.

[92] Lazaridou A, Martel MO, Cornelius M (2019). The association between daily physical activity and pain among patients with knee osteoarthritis: the moderating role of pain catastrophizing. Pain Med.

[93] Tighe CA, Youk A, Ibrahim SA (2020). Pain catastrophizing and arthritis self-efficacy as mediators of sleep disturbance and osteoarthritis symptom severity. Pain Med.

[94] Hayashi K, Morishima T, Ikemoto T (2019). Pain catastrophizing is independently associated with quality of life in patients with severe hip osteoarthritis. Pain Med.

[95] Helminen EE, Arokoski JPA, Selander TA, Sinikallio SH (2020). Multiple psychological factors predict pain and disability among community-dwelling knee osteoarthritis patients: a five-year prospective study. Clin Rehabil.

[96] Sinikallio SH, Helminen EE, Valjakka AL (2014). Multiple psychological factors are associated with poorer functioning in a sample of community-dwelling knee osteoarthritis patients. J Clin Rheumatol.

[97] Nishigami T, Tanaka S, Mibu A (2021). Knee-related disability was largely influenced by cognitive factors and disturbed body perception in knee osteoarthritis. Sci Rep.

[98] Keefe FJ, Lefebvre JC, Egert JR (2000). The relationship of gender to pain, pain behavior, and disability in osteoarthritis patients: the role of catastrophizing. Pain.

[99] France CR, Keefe FJ, Emery CF (2004). Laboratory pain perception and clinical pain in post-menopausal women and age-matched men with osteoarthritis: relationship to pain coping and hormonal status. Pain.

[100] Somers TJ, Keefe FJ, Carson JW (2008). Pain catastrophizing in borderline morbidly obese and morbidly obese individuals with osteoarthritic knee pain. Pain Res Manag J Can Pain Soc.

[101] Odole A, Ekediegwu E, Ekechukwu END (2022). Chronic knee osteoarthritis: relationships of body mass index and selected psychosocial factors among Nigerians. Hong Kong Physiother J.

[102] Ikemoto T, Miyagawa H, Shiro Y (2017). Relationship between biological factors and catastrophizing and clinical outcomes for female patients with knee osteoarthritis. World J Orthop.

[103] Aily JB, de Almeida AC, Ramírez PC (2021). Lower education is an associated factor with the combination of pain catastrophizing and kinesiophobia in patients with knee osteoarthritis?. Clin Rheumatol.

[104] Kubo M, Maeda T, Kumagai K (2022). Phenotypic classification of knee osteoarthritis according to pain mechanisms; a clinical observational study. J Orthop Sci.

[105] Mulrooney E, Neogi T, Dagfinrud H (2022). The associations of psychological symptoms and cognitive patterns with pain and pain sensitization in people with hand osteoarthritis. Osteoarthr Cartil Open.

[106] Nemati D, Quintero D, Best TM, Kaushal N (2023). Investigating the association between knee osteoarthritis symptoms with pain catastrophizing domains between Hispanics and non-Hispanic Whites. Rheumatol Int.

[107] Fullwood D, Gomez RN, Huo Z (2021). A mediation appraisal of catastrophizing, pain-related outcomes, and race in adults with knee osteoarthritis. J Pain.

[108] Jones AC, Kwoh CK, Groeneveld PW (2008). Investigating racial differences in coping with chronic osteoarthritis pain. J Cross Cult Gerontol.

[109] Uritani D, Campbell PK, Metcalf B, Egerton T (2022). A comparison of psychological characteristics in people with knee osteoarthritis from Japan and Australia: a cross-sectional study. PLoS ONE.

[110] Van Denburg AN, Shelby RA, Caldwell DS (2018). Self-efficacy for pain communication moderates the relation between ambivalence over emotional expression and pain catastrophizing among patients with osteoarthritis. J Pain.

[111] Booker SQ, Sibille KT, Terry EL (2020). Psychological predictors of perceived age and chronic pain impact in individuals with and without knee osteoarthritis. Clin J Pain.

[112] Wang Y, Li X, Zhang Y (2023). Association of sleep disturbance with catastrophizing and knee pain: data from the osteoarthritis initiative. Arthritis Care Res (Hoboken).

[113] Keefe FJ, Affleck G, France CR (2004). Gender differences in pain, coping, and mood in individuals having osteoarthritic knee pain: a within-day analysis. Pain.

[114] Nian X, He Y, Ji Y (2019). Associations between pain patterns and self-reported clinical outcomes in patients with knee osteoarthritis. Pain Med.

[115] Tanaka S, Nishigami T, Ohishi K (2021). “But it feels swollen!”: the frequency and clinical characteristics of people with knee osteoarthritis who report subjective knee swelling in the absence of objective swelling. Pain Rep.

[116] El-Najjar AR, Abdelwhab SM, Elsammak Ahmad A (2020). Potential role of brain biomarkers in primary knee osteoarthritis patients using magnetic resonance spectroscopy. Egypt Rheumatol.

[117] Terry EL, Tanner JJ, Cardoso JS (2021). Associations of pain catastrophizing with pain-related brain structure in individuals with or at risk for knee osteoarthritis: sociodemographic considerations. Brain Imaging Behav.

[118] Terry EL, Tanner JJ, Cardoso JS (2022). Associations between pain catastrophizing and resting-state functional brain connectivity: ethnic/race group differences in persons with chronic knee pain. J Neurosci Res.

[119] Wade JB, Riddle DL, Price DD, Dumenci L (2011). Role of pain catastrophizing during pain processing in a cohort of patients with chronic and severe arthritic knee pain. Pain.

[120] Willett MJ, Siebertz M, Petzke F (2020). The extent of pain is associated with signs of central sensitization in patients with hip osteoarthritis. Pain Pract.

[121] Gür O, Başar S, Esen E (2021). The relationship of kinesiophobia and pain catastrophizing with pain, range of motion, muscle strength and function in osteoarthritis. Int J Disabil Sports Health Sci.

[122] Hoogendam L, van der Oest MJW, Tsehaie J (2021). Psychological factors are more strongly associated with pain than radiographic severity in non-invasively treated first carpometacarpal osteoarthritis. Disabil Rehabil.

[123] Fu K, Metcalf B, Bennell KL (2021). The association between psychological factors and pain exacerbations in hip osteoarthritis. Rheumatology (Oxford).

[124] Tanaka R, Hirohama K, Kurashige Y (2020). Prediction models considering psychological factors to identify pain relief in conservative treatment of people with knee osteoarthritis: A multicenter, prospective cohort study. J Orthop Sci.

[125] Rayahin JE, Chmiel JS, Hayes KW (2014). Factors associated with pain experience outcome in knee osteoarthritis. Arthritis Care Res (Hoboken).

[126] Alschuler KN, Molton IR, Jensen MP, Riddle DL (2013). Prognostic value of coping strategies in a community-based sample of persons with chronic symptomatic knee osteoarthritis. Pain.

[127] Frey-Law LA, Bohr NL, Sluka KA (2016). Pain sensitivity profiles in patients with advanced knee osteoarthritis. Pain.

[128] Tanaka R, Hirohama K (2018). Association of pain quality with pain catastrophizing and self-efficacy in people with knee osteoarthritis. Prog Rehabil Med.

[129] Gandhi R, Tsvetkov D, Dhottar H (2010). Quantifying the pain experience in hip and knee osteoarthritis. Pain Res Manag.

[130] López-Bravo MD, Zamarrón-Cassinello MD, La TR (2021). Psychological factors associated with functional disability in patients with hip and knee osteoarthritis. Behav Med (Washington, DC).

[131] Helminen EE, Sinikallio SH, Valjakka AL (2016). Determinants of pain and functioning in knee osteoarthritis: a one-year prospective study. Clin Rehabil.

[132] López-López A, Montorio I, Izal M, Velasco L (2008). The role of psychological variables in explaining depression in older people with chronic pain. Aging Ment Health.

[133] Terry EL, Fullwood MD, Booker SQ (2020). Everyday discrimination in adults with knee pain: the role of perceived stress and pain catastrophizing. J Pain Res.

[134] Zhao R, Ji Y, Li J (2022). Pain empathy and its association with the clinical pain in knee osteoarthritis patients. J Pain Res.

[135] Goodin BR, Glover TL, Sotolongo A (2013). The association of greater dispositional optimism with less endogenous pain facilitation is indirectly transmitted through lower levels of pain catastrophizing. J Pain.

[136] Porter LS, Keefe FJ, Wellington C, De Williams A (2008). Pain communication in the context of osteoarthritis: patient and partner self-efficacy for pain communication and holding back from discussion of pain and arthritis-related concerns. Clin J Pain.

[137] Martire LM, Zhaoyang R, Marini CM (2019). Daily and bidirectional linkages between pain catastrophizing and spouse responses. Pain.

[138] Carriere JS, Lazaridou A, Martel MO (2020). The moderating role of pain catastrophizing on the relationship between partner support and pain intensity: a daily diary study in patients with knee osteoarthritis. J Behav Med.

[139] Song J, Dunlop DD, Semanik PA (2018). Reallocating time spent in sleep, sedentary behavior and physical activity and its association with pain: a pilot sleep study from the osteoarthritis initiative. Osteoarthr Cartil.

[140] Tang HY, McCurry SM, Pike KC (2017). Differential predictors of nighttime and daytime sleep complaints in older adults with comorbid insomnia and osteoarthritis pain. J Psychosom Res.

[141] Campbell CM, Buenaver LF, Finan P (2015). Sleep, pain catastrophizing, and central sensitization in knee osteoarthritis patients with and without insomnia. Arthritis Care Res (Hoboken).

[142] Vitiello MV, McCurry SM, Shortreed SM (2014). Short-term improvement in insomnia symptoms predicts long-term improvements in sleep, pain, and fatigue in older adults with comorbid osteoarthritis and insomnia. Pain.

[143] Lerman SF, Finan PH, Smith MT, Haythornthwaite JA (2017). Psychological interventions that target sleep reduce pain-catastrophizing in knee osteoarthritis. Pain.

[144] Barbosa SP, Marques L, Sugawara A (2022). Predictors of the health-related quality of life (HRQOL) in SF-36 in knee osteoarthritis patients: a multimodal model with moderators and mediators. Cureus.

[145] Hidaka R, Tanaka T, Hashikura K (2023). Association of high kinesiophobia and pain catastrophizing with quality of life in severe hip osteoarthritis: a cross-sectional study. BMC Musculoskelet Disord.

[146] Hopman-Rock M, Kraaimaat FW, Odding E, Bijlsma JW (1998). Coping with pain in the hip or knee in relation to physical disability in community-living elderly people. Arthritis Care Res.

[147] Uritani D, Kasza J, Campbell PK (2020). The association between psychological characteristics and physical activity levels in people with knee osteoarthritis: a cross-sectional analysis. BMC Musculoskelet Disord.

[148] Wideman TH, Finan PH, Edwards RR (2014). Increased sensitivity to physical activity among individuals with knee osteoarthritis: relation to pain outcomes, psychological factors, and responses to quantitative sensory testing. Pain.

[149] Zhaoyang R, Martire LM, Darnall BD (2020). Daily pain catastrophizing predicts less physical activity and more sedentary behavior in older adults with osteoarthritis. Pain.

[150] Kopp B, Furlough K, Goldberg T (2021). Factors associated with pain intensity and magnitude of limitations among people with hip and knee arthritis. J Orthop.

[151] Furlough K, Miner H, Crijns TJ (2021). What factors are associated with perceived disease onset in patients with hip and knee osteoarthritis?. J Orthop.

[152] Tanaka R, Hirohama K, Ozawa J (2019). Can muscle weakness and disability influence the relationship between pain catastrophizing and pain worsening in patients with knee osteoarthritis? A cross-sectional study. Braz J Phys Ther.

[153] Baert IAC, Meeus M, Mahmoudian A (2017). Do psychosocial factors predict muscle strength, pain, or physical performance in patients with knee osteoarthritis?. J Clin Rheumatol.

[154] Sánchez-Herán Á, Agudo-Carmona D, Ferrer-Peña R (2016). Postural stability in osteoarthritis of the knee and hip: analysis of association with pain catastrophizing and fear-avoidance beliefs. PM R.

[155] Fidelis-de-Paula-Gomes CA, Dibai-Filho AV, Ferreira CSB (2022). Correlation among pain intensity, catastrophizing, and falls in older individuals with unilateral knee osteoarthritis: a cross-sectional study. J Manip Physiol Ther.

[156] Gomes CAFP, Dibai-Filho AV, Biasotto-Gonzalez DA (2018). Association of pain catastrophizing with static balance, mobility, or functional capacity in patients with knee osteoarthritis: a blind cross-sectional study. J Manip Physiol Ther.

[157] Somers TJ, Keefe FJ, Pells JJ (2009). Pain catastrophizing and pain-related fear in osteoarthritis patients: relationships to pain and disability. J Pain Symptom Manag.

[158] White DK, Tudor-Locke C, Zhang Y (2016). Prospective change in daily walking over two years in older adults with or at risk of knee osteoarthritis: the MOST Study. Osteoarthr Cartil.

[159] Suzuki Y, Iijima H, Aoyama T (2020). Pain catastrophizing affects stair climbing ability in individuals with knee osteoarthritis. Clin Rheumatol.

[160] Lecorney J, Verhoeven F, Chouk M (2018). Correlation between catastrophizing and Lequesne index in case of osteoarthritis of the knee: a prospective study. Jt Bone Spine.

[161] Rini C, Katz AWK, Nwadugbo A (2021). Changes in identification of possible pain coping strategies by people with osteoarthritis who complete web-based pain coping skills training. Int J Behav Med.

[162] Somers TJ, Blumenthal JA, Guilak F (2012). Pain coping skills training and lifestyle behavioral weight management in patients with knee osteoarthritis: a randomized-controlled study. Pain.

[163] Allen KD, Somers TJ, Campbell LC (2019). Pain coping skills training for African Americans with osteoarthritis: results of a randomized controlled trial. Pain.

[164] Taylor SS, Oddone EZ, Coffman CJ (2018). Cognitive mediators of change in physical functioning in response to a multifaceted intervention for managing osteoarthritis. Int J Behav Med.

[165] Shelby RA, Somers TJ, Keefe FJ (2008). Domain specific self-efficacy mediates the impact of pain catastrophizing on pain and disability in overweight and obese osteoarthritis patients. J Pain.

[166] Youngcharoen P, Saraboon Y, Aree-Ue S (2020). Factors influencing health status in older people with knee osteoarthritis. Jpn J Nurs Sci.

[167] McKnight PE, Afram A, Kashdan TB (2010). Coping self-efficacy as a mediator between catastrophizing and physical functioning: treatment target selection in an osteoarthritis sample. J Behav Med.

[168] de Almeida AC, Aily JB, Pedroso MG (2021). Reductions of cardiovascular and metabolic risk factors after a 14-week periodized training model in patients with knee osteoarthritis: a randomized controlled trial. Clin Rheumatol.

[169] Rabiei P, Sheikhi B, Letafatkar A (2023). Examining the influence of pain neuroscience education followed by a Pilates exercises program in individuals with knee osteoarthritis: a pilot randomized controlled trial. Arthritis Res Ther.

[170] Öztürk Ö, Bombacı H, Keçeci T, Algun ZC (2021). Effects of additional action observation to an exercise program in patients with chronic pain due to knee osteoarthritis: a randomized-controlled trial. Musculoskelet Sci Pract.

[171] Supe HM, Mungikar SS, Katage GA (2023). Effect of pain neuroscience education with conventional physiotherapy via telerehabilitation on pain catastrophizing and function in patients with osteoarthritis knee: a randomized controlled trial. J Midlife Health.

[172] Öztürk Ö, Algun ZC, Bombacı H, Erdoğan SB (2021). Changes in prefrontal cortex activation with exercise in knee osteoarthritis patients with chronic pain: an fNIRS study. J Clin Neurosci.

[173] Uckun AC, Donmez BK, Yurdakul FG (2020). The role of pain catastrophizing and depression in the outcomes of physical therapy in a prospective osteoarthritis cohort. Pain Physician.

