# Supplementary material for: Pain catastrophizing in rheumatic diseases: prevalence, origin, and implications
Source: Rheumatol Int. 2024 Apr 12;44(6):985–1002. doi: 10.1007/s00296-024-05583-8 (PMC11108955; doi:10.1007/s00296-024-05583-8)
Supplement: Supplementary file 1 — Supplementary file1 (DOCX 20 KB) [file 296_2024_5583_MOESM1_ESM.docx]

**Appendix 1.** Specific description of keyword strings utilized in performing literature search process in PubMED/MEDLINE database.

1. Rheumatoid arthritis

(“catastrophization” [MeSH Terms] OR catastrophi* [tw]) AND “pain*” AND “rheumatoid arthritis” AND English [la]

1. Psoriatic arthritis

(“catastrophization” [MeSH Terms] OR catastrophi* [tw]) AND “pain*” AND “psoriatic arthritis” AND English [la]

1. Axial spondylarthritis

("catastrophization" [MeSH Terms] or catastrophi* [tw]) AND "pain*" and ("Axial" OR "Ankylosing") and English [la]

1. Systemic lupus erythematosus

("catastrophization" [MeSH Terms] or catastrophi* [tw]) AND "pain*" and ("SLE" OR "Lupus") and English [la]

1. Systemic sclerosis

(“catastrophization” [MeSH Terms] OR catastrophi* [tw]) AND “pain*” AND “systemic sclerosis” AND English [la]

1. Sjogren syndrome

("catastrophization" [MeSH Terms] or catastrophi* [tw]) AND "pain*" and ("sjogren" OR "Sjögren") and English [la]

1. Osteoarthritis

("catastrophization" [MeSH Terms] or catastrophi* [tw]) AND "pain*" and ("Osteoarthritis") and English [la]

1. Juvenile idiopathic arthritis

(“catastrophization” [MeSH Terms] OR catastrophi* [tw]) AND “pain*” AND “juvenile” AND “arthritis” AND English [la]

**Appendix 2.** Specific description of keyword strings utilized in performing literature search process in Scopus database.

1. Rheumatoid arthritis

TITLE-ABS-KEY ( catastrophiz*  OR  "pain catastrophizing"  OR  "catastrophic thinking"  OR  "Rumination" )  AND  TITLE-ABS-KEY ( pain )  AND  TITLE-ABS-KEY ( rheumatoid  AND arthritis )  AND  ( LIMIT-TO ( LANGUAGE ,  "English" ) )

1. Psoriatic arthritis

TITLE-ABS-KEY ( catastrophiz*  OR  "Pain Catastrophizing"  OR  "catastrophic thinking"  OR  "Rumination" )  AND  TITLE-ABS-KEY ( pain )  AND  TITLE-ABS-KEY ( Psoriatic  AND arthritis )  AND  ( LIMIT-TO ( LANGUAGE ,  "English" ) )

1. Axial spondylarthritis

TITLE-ABS-KEY ( catastrophiz*  OR  "Pain Catastrophizing"  OR  "catastrophic thinking"  OR  "Rumination" )  AND  TITLE-ABS-KEY ( pain )  AND  TITLE-ABS-KEY ( axial  OR ankylosing )  AND  ( LIMIT-TO ( LANGUAGE ,  "English" ) )

1. Systemic lupus erythematosus

TITLE-ABS-KEY ( catastrophiz*  OR  "Pain Catastrophizing"  OR  "catastrophic thinking"  OR  "Rumination" )  AND  TITLE-ABS-KEY ( pain )  AND  TITLE-ABS-KEY ( SLE  OR Lupus )  AND  ( LIMIT-TO ( LANGUAGE ,  "English" ) )

1. Systemic sclerosis

TITLE-ABS-KEY ( catastrophiz*  OR  "Pain Catastrophizing"  OR  "catastrophic thinking"  OR  "Rumination" )  AND  TITLE-ABS-KEY ( pain )  AND  TITLE-ABS-KEY ( systemic  AND sclerosis )  AND  ( LIMIT-TO ( LANGUAGE ,  "English" ) )

1. Sjogren syndrome

TITLE-ABS-KEY ( catastrophiz*  OR  "Pain Catastrophizing"  OR  "catastrophic thinking"  OR  "Rumination" )  AND  TITLE-ABS-KEY ( pain )  AND  TITLE-ABS-KEY ( Sjogren or Sjögren)  AND  ( LIMIT-TO ( LANGUAGE ,  "English" ) )

1. Osteoarthritis

TITLE-ABS-KEY ( catastrophiz*  OR  "Pain Catastrophizing"  OR  "catastrophic thinking"  OR  "Rumination" )  AND  TITLE-ABS-KEY ( pain )  AND  TITLE-ABS-KEY ( osteoarthritis)  AND  ( LIMIT-TO ( LANGUAGE ,  "English" ) )

1. Juvenile idiopathic arthritis

TITLE-ABS-KEY ( catastrophiz*  OR  "Pain Catastrophizing"  OR  "catastrophic thinking"  OR  "Rumination" )  AND  TITLE-ABS-KEY ( pain )  AND  TITLE-ABS-KEY ( juvenile  AND arthritis )  AND  ( LIMIT-TO ( LANGUAGE ,  "English" ) )
